# Visual evoked potential in generalized joint hypermobility: A case–control study

**DOI:** 10.1002/brb3.3493

**Published:** 2024-04-19

**Authors:** Leila Sadat Mohamadi Jahromi, Amin Sayyadi, Aida Askarian, Alireza Dabbaghmanesh, Sharareh Roshanzamir

**Affiliations:** ^1^ Department of Physical Medicine and Rehabilitation, Shiraz Medical School Shiraz University of Medical Sciences Shiraz Iran; ^2^ Shiraz Medical School Shiraz University of Medical Sciences (SUMS) Shiraz Iran; ^3^ Department of Internal Medicine, Shiraz Medical School Shiraz University of Medical Sciences (SUMS) Shiraz Iran

**Keywords:** Ehlers–Danlos syndrome, generalized joint hypermobility, nerve conduction, visual evoked potential

## Abstract

**Introduction:**

Generalized joint hypermobility (GJH) can be the result of several hereditary connective tissue disorders, especially Ehlers–Danlos syndrome. Cerebrovascular manifestations are among the most common complications in this disorder, and understanding their extent can help better diagnosis and prevention of hazardous events. We investigated visual evoked potential (VEP) changes in patients with GJH and compared them with healthy individuals.

**Methods:**

Our case–control study included 90 patients who fulfilled the Beighton score (B score) for joint hypermobility and other 90 healthy participants. All of them went under VEP study, and the amplitude and latency of the evoked potential (P100) were compared to each other.

**Results:**

The Case group had significantly higher B score (7.18 ± 0.967 vs. 1.18 ± 0.712), P100 latency (110.23 ± 6.64 ms vs. 100.18 ± 4.273 ms), and amplitude (6.54 ± 1.26 mv vs. 6.50 ± 1.29 mv) compared with the Control group, but the difference was only significant regarding B score, and P100 latency (*p*‐value <.0001). Moreover, both latency and amplitude of P100 had significantly positive correlations with the B score in the Case group (*p*‐value <.0001), but such correlations were not found in the Control group (*p*‐value = .059).

**Conclusion:**

Our study could reveal VEP changes, especially significant P100 latency in GJH patients without previous neurologic or musculoskeletal disorders. Whether these changes are due to GJH itself or are predictive of inevitable neurologic disease or visual pathway involvement, particularly Multiple Sclerosis needs further investigation with longer follow‐up periods.

## Graphical abstract

Our case–control study comprised performing visual evoked potential studies on 90 patients who fulfilled the Beighton score (B score) for joint hypermobility and other 90 healthy participants. Our study could reveal VEP changes, especially significant P100 latency in GJH patients without previous neurologic or musculoskeletal disorders.

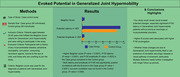

## 1 LIMITATIONS

Some limitations of this study are inherited in case–control studies, including its retrospective nature and recall bias. Having a small sample size, lack of long‐term follow‐up for our cases, and not performing laboratory workup or skin biopsy for definite diagnosis of collagen disorders were other limitations. Additionally, current knowledge regarding the VEP changes in GJH is scarce which made comparing the results difficult.

## 2 INTRODUCTION

Joint hypermobility is the excess range of motion for a particular joint. Generalized joint hypermobility (GJH) is the occurrence of joint hypermobility in several joints in the body (Parks et al., 2023). Many individuals with GJH are asymptomatic and even use their flexibility to their advantage in sports, but some may experience pain related to their hypermobility state. GJH is the feature of multiple conditions such as hypermobility spectrum disorder (HSD) and Ehlers–Danlos syndrome (EDS) but conflicts remain regarding the cause of GJH. Although multiple hereditary connective tissue disorders, including EDS, Marfan, and osteogenesis imperfect, can be the roots, most patients have not identified hereditary connective tissue disorder (Boudreau et al., 2020; Henderson et al., 2017; Kose Ozlece et al., 2015). The exact prevalence is not known, but a study done in Wales, United Kingdom reported a prevalence of 194.2 per 100,000 in 2016/2017 for either EDS or HSD (Demmler et al., 2019). Reporting on different ages, sexes, and ethnicities and using varied methodologies are the main roots of this variance. The greater severity of the joint laxity occurs during younger ages, whereas it begins to decrease with the aging process (Boudreau et al., 2020).

The diagnosis of GJH is based on clinical features according to the Beighton score (B score). B score is a scaling system usually used for diagnosis of GJH to understand the extend of the hypermobility in multiple predetermined joints (Castori & Voermans, 2014).

EDS is an important cause of GJH, and besides its principal features, this disorder can be accompanied by several neurological manifestations with fatigue and chronic pain, especially headache, being the most common neurological symptoms. EDS also causes abnormalities in different components of the visual system such as cornea surface irregularity (Villani et al., 2013) and conjunctivochalasis (Whitaker et al., 2012). EDS is sometimes accompanied by other disorders. Few studies (Kose Ozlece et al., 2015; Vilisaar et al., 2008) have noted this concomitance with multiple sclerosis (MS) and even reported that the prevalence of MS is 10‐11 times more in EDS patients than the general population. This can be the result of multiple factors, including MS‐EDS, associated gene mutations and defects of the EDS proteins made by oligodendrocytes and astrocytes in the central nervous system affecting collagens in the level of vascular walls. They can cause myelin destruction and inflammation secondary to the increase in the migration of the immune cells to the central nervous system (Kose Ozlece et al., 2015).

Visual evoked potential (VEP) is recording the visual pathway electrical activity from the calcarine cortex to the optic nerve (Baiano & Zeppieri, 2023). Since having a healthy functional visual pathway from the retina to the cortex is essential for a normal VEP, demyelinating disorders can cause amplitude and latency abnormalities (Steczkowska et al., 2009; Tsoumanis et al., 2023).

Considering the relatively high prevalence of GJH and the importance of neurological and visual defects, understanding the extent and pattern of nervous system involvement can help clinicians in the early detection and prevention of subsequent catastrophic complications. Additionally, to the best of our knowledge, no study has investigated VEP alterations in GJH patients with no previous neurologic or musculoskeletal disease. Thus, we conducted a case–control study to evaluate VEP abnormalities in patients with GJH compared with healthy individuals.

## 3 MATERIALS AND METHODS

### 3.1 Participants and procedures

In this case–control study, 90 patients with diagnosed GJH (Case group) and 90 healthy individuals without this condition (Control group) were evaluated. All of them went under VEP study. We compared recorded parameters including amplitude and latency of the evoked potential between two groups.

B score was used for the diagnosis of patients with GJH. This scoring system is based on the nine movements each adding one point to the total score. The person is considered to have GJH if the score is four or greater (Table 1) (Malek et al., 2021).

**TABLE 1:** Beighton score for joint hypermobility syndrome.

Test	Side	Points
Passive dorsiflexion of the fifth metacarpophalangeal joint to ≥90 degrees	Right	1
	Left	1
Passive hyperextension of the knee ≥10 degrees	Right	1
	Left	1
Passive apposition of the thumb to the flexor side of the forearm; although shoulder is flexed 90 degrees, elbow is extended, and hand is pronated	Right	1
	Left	1
Forward flexion of the trunk, with the knees straight, so that the hand palms rest easily on the floor		1
Total		9

Patients aged between 20 and 45 years that fulfilled the abovementioned criteria for GJH and were willing to participate in the study were included. Participants with a history of any musculoskeletal disease, peripheral neuropathy, convulsion, consuming neurotoxic agents, trauma to the eye or orbit, and those who are unwilling to join the study were excluded.

For performing VEP, we asked the individual to sit on a chair positioned one meter away from the monitor in a dedicated VEP room. Following the abrasion of the scalp skin, we positioned the active electrode at the OZ point (10% above the inion point on the line between inion and nasion), the reference electrode at the CZ point (the intersection of the line connecting both ears and the line connecting the inion and the nasion), and the ground electrode on the frontal bone. We instructed the patient to cover one eye with her hand while keeping the other eye open and focused on the front screen. In VEP, both the amplitude and latency of positive and negative waves were typically evaluated. Latency, defined as the time required for stimulation of the nerve terminal to reach maximum response, was less influenced by external factors and therefore considered the most important measurement parameter. A peak latency of the positive component of the evoked potential less than 100 ms, abbreviated as P100, was considered normal, whereas a latency exceeding 120 ms would be deemed abnormal (DeLisa et al., 2005). Due to the wide range of amplitude variations observed, it was not considered a reliable parameter in patients.

### 3.2 Statistical analysis

SPSS version 13 was our statistical analysis software. We used the Mann–Whitney *U* test, chi‐squared test, independent *T*‐test, and Pearson's linear regression for data analysis. The *p*‐value <.05 was considered statistically significant.

### 3.3 Ethical considerations

The written informed consent was obtained from each participant after describing all the steps and purposes of this study. This study was performed according to the Declaration of Helsinki, and the medical Ethics Committee of Shiraz University of Medical Sciences approved this study with code number “IR.SUMS.REC.1394.S789.”

## 4 RESULTS

Most participants were females in both case (52[57.8%] vs. 38[42.2%]) and control (49[54.4%] vs. 41[45.6%]) groups; additionally, the Mean ± SD of age in the case and control group was 26.89 ± 5.29 and 27.34 ± 5.93 years, respectively. Case and control groups were comparable in terms of sex (*p*‐value = .764) and age (*p*‐value = .802).

**FIGURE 1:**
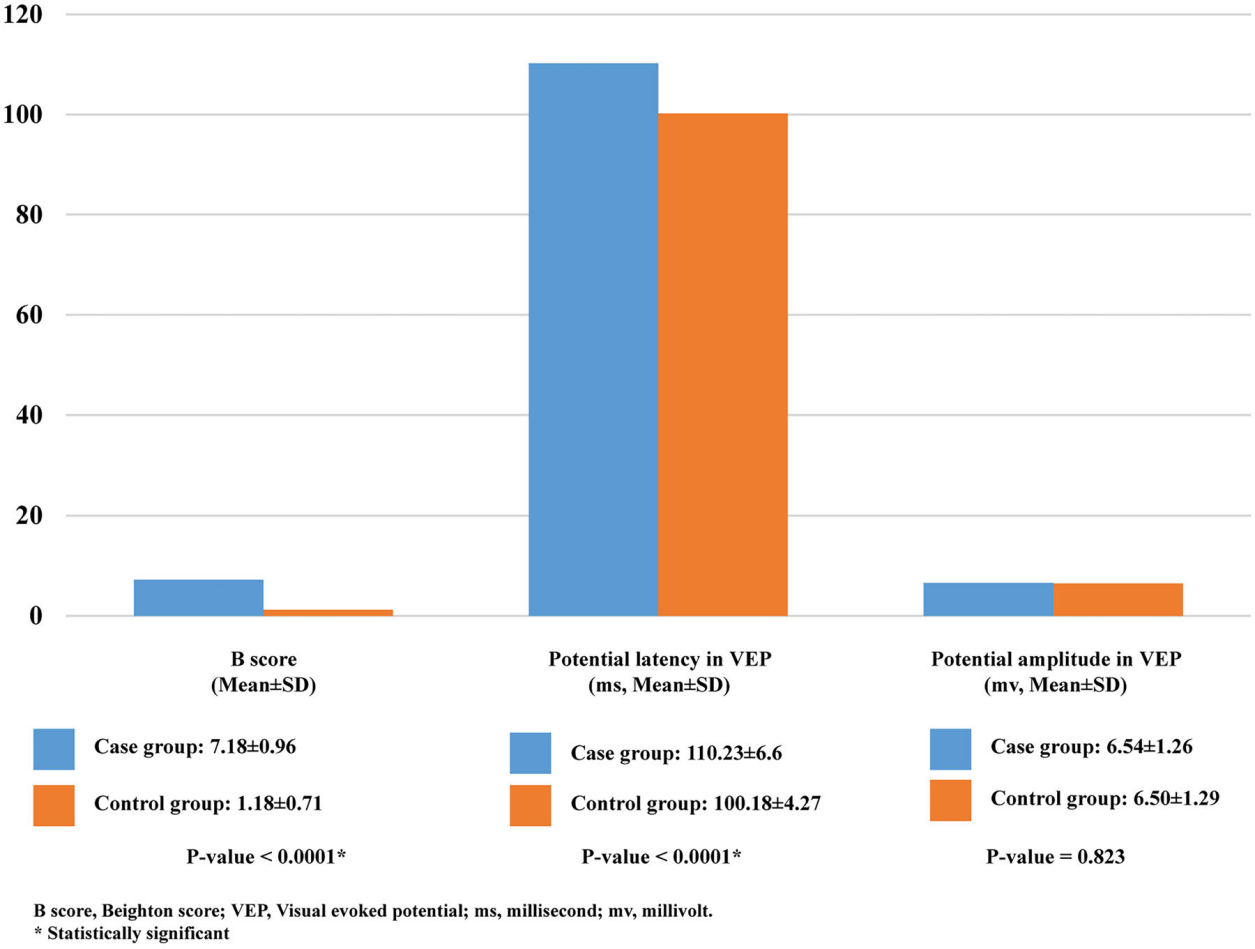
Baseline characteristics of participants.

Mean ± SD for both B score (7.18 ± 0.967 vs. 1.18 ± 0.712) and latency (110.23 ± 6.64 ms vs. 100.18 ± 4.27 3 ms) were significantly higher in the case group (*p*‐value <.0001) compared to the control group. Although the case group had a higher amplitude than the control group (6.54 ± 1.26 mv vs. 6.50 ± 1.29 mv), the difference was not statistically significant (*p*‐value = .823) (Figure 1).

In the case group, both correlations were significantly positive (B score and latency: *p*‐value <.0001, *r* = .445; B score and amplitude: *p*‐value = .017, *r* = .251). Such significant correlations were not observed in the control group (B score and latency: *p*‐value = .059, *r* = .2; B score and amplitude: *p*‐value = .75, *r* = −.034) (Table 2).

**TABLE 2:** Correlation between visual evoked potential (VEP) parameters and Beighton score (B score) in both case and control groups.

Correlations	Case		Control	
	*p*‐Value	*r*	*p*‐Value	*r*
B score and latency	<.0001	.445	.059	.2
B score and amplitude	.017	.251	.75	−.034

Abbreviations: B score, Beighton score; *r*, Pearson correlation coefficient; VEP, visual evoked potential.

## 5 DISCUSSION

This case–control study was conducted to investigate VEP prevalence and pattern in individuals with GJH. Both latency and amplitude of VEP were higher in the case group compared with the control group, but the difference was only significant regarding the latency of VEP. Additionally, both latency and amplitude had significantly positive correlations with the B score in the case group, but such correlations were not found in the control group.

Most individuals with GJH do not have a hereditary connective tissue disorder. However, GJH can be associated with several hereditary connective tissue disorders, including EDS, Marfan syndrome, and osteogenesis imperfecta (Boudreau et al., 2020; Henderson et al., 2017; Kose Ozlece et al., 2015).

The data regarding the alterations of VEP in GJH and its related pathologies is scarce. Maestrini et al. (2023) performed a case–control study to investigate whether migraine (MIG)‐induced VEP changes are present in EDS or not. Their study consisted of three groups: 22 hereditary EDS (hEDS) patients with MIG, 22 non‐hEDS patients with MIG, and 22 healthy controls (HC). Although P100 latency was higher in MIG (116.55 ± 5.13 ms) than in hEDS (115.45 ± 5.83 ms) and HC (112.95 ± 5.54 ms), no significant difference was observed (*p*‐value >.05). While evaluating amplitude, they recognized that hEDS group had the lowest N75‐P100 (7.27 ± 3.35 mv) and P100‐N145 (5.35 ± 3.75 mv) amplitudes compared with MIG (N75‐P100: 8.61 ± 3.38 mv, P100‐N145: 5.56 ± 2.24 mv) and HC (N75‐P100: 9.02 ± 0.61 mv, P100‐N145: 12.47 ± 1.06 mv). The between group difference was significant in P100‐N145.

Mathew et al. (2005) mentioned four patients with EDS. Two of them had VEP changes: first, a 20‐year‐old male with delayed motor milestones and unsteady gait from childhood; they considered EDS due to hyperextensible joints, hyperelastic skin, primary optic atrophy and sensorineural deafness, distal limb weakness; nerve conduction study implied evidence of demyelinating motor‐sensory neuropathy; magnetic resonance imaging (MRI) was normal; VEP was bilaterally absent; sural nerve biopsy revealed focal loss of large diameter fibers and demyelination; second, a 22‐year‐old male with swaying while walking, slurring of speech, involuntary movements, and fatigability; his nerve conduction study was normal; MRI revealed cerebellar and brainstem atrophy; his VEP did not show any abnormality. Kose Ozlece et al. (2015) reported a 26‐year‐old male with complaints of numbness and weakness in his left arm and leg and low vision in his left eye; he was diagnosed with EDS for 10 years; his cranial and cervical MRI revealed multiple demyelinating plaques; in VEP, they noticed bilateral prolonged P100 latency; they diagnosed him with simultaneous EDS and MS.

VEP abnormalities are typically found in demyelinating disorders, particularly MS. Increased latency, when the waveform is preserved implies a demyelinating process. VEP has two main diagnostic applications in MS: confirming the presence of visual pathology and detecting pathologic processes in asymptomatic patients. Nikolic et al. (2022) evaluated the significance of VEP in diagnosing optic neuritis and clinically silent lesions in 52 pediatric patients with MS. They found that 40 patients (76.9%) had Pathological VEP findings, and 22 patients (42.3%) had clinically silent lesions; patients with MS had significantly higher P100 wave prolongation compared to controls. They concluded that VEP is a fast and accessible method for detecting clinical and subclinical lesions, and prolonged P100 latency could be considered the main indicator of optic neuritis. Voitenkov et al. (2015) worked on VEP in children with clinically isolated syndromes which later proved to be MS onset. Their study consisted of 47 patients and 30 healthy participants. Delayed VEP was found in 58% of patients, even in those without signs of retrobulbar neuritis; P100 amplitude drops in 29% of patients. They believed that VEP is effective in early diagnosis of MS.

VEP parameters also fluctuate with time; Chatziralli et al. (2012) evaluated visual acuity, and VEP changes in 23 patients who suffered optic neuritis due to MS. They reported that visual acuity improved over time; additionally, both P100 amplitude and latency improved significantly compared to controls in follow‐up (*p*‐value <.0001).

To the best of our knowledge, this is the first study evaluating VEP changes in GJH patients without neurological or musculoskeletal disorders. Although our study could reveal VEP changes in GJH patients, due to lack of follow‐up we could not investigate whether these changes are inherited in the GJH or are predictive of neurological diseases, especially MS. Thus, performing similar studies with follow‐up seems to be of value.

Although studies have mentioned multiple mechanisms for explaining the underlying pathophysiology of EDS‐MS association, the exact mechanism is yet to be discovered. Most hypotheses are related to extracellular matrix (ECM) proteins and their interactions in central nervous system inflammation and demyelination. It seems that EDS is associated with mutations in ECM proteins. EDS is also related to synthesis defects in the cells involved in cell migration and organization. In the central nervous system, oligodendrocytes and astrocytes produce ECM proteins and are associated with astroglial response in MS lesions. A hypothesis is that ECM proteins that are present in the blood vessel walls such as collagen and tenascin increase immune cell migration to the central nervous system and subsequently cause myelin destruction. Another mentioned hypothesis is regarding a gene; this hypothesis indicates that there may be a polygenic effect in MS, and EDS is the result of one of these mutations (Kose Ozlece et al., 2015; Vilisaar et al., 2008).

## 6 CONCLUSION

In our study, the latency of P100 was significantly higher in patients with GJH compared with healthy individuals. Additionally, higher B scores were associated with higher P100 prolongation. Our study could reveal VEP changes, especially significant P100 latency in GJH patients without previous neurologic or musculoskeletal disorders. Whether these changes are due to GJH itself or are predictive of inevitable neurologic disease or visual pathway involvement, particularly MS needs further investigation with longer follow‐up periods.

## AUTHOR CONTRIBUTIONS

**Leila Sadat Mohamadi Jahromi**: Conceptualization; investigation; methodology; writing—original draft; writing—review and editing. **Amin Sayyadi**: Formal analysis; software; visualization; writing—original draft; writing—review and editing. **Aida Askarian**: Data curation; formal analysis; investigation. **Alireza Dabbaghmanesh**: Investigation; methodology. **Sharareh Roshanzamir**: Conceptualization; methodology; project administration; supervision; writing—review and editing.

## CONFLICT OF INTEREST STATEMENT

No potential conflicts of interest were reported by the authors.

## FUNDING INFORMATION

The authors received no financial support for the research, authorship or publication of this article.

## PATIENT CONSENT STATEMENT

The written informed consent was obtained from each participant after describing all steps of this study and the purposes for them.

### PEER REVIEW

The peer review history for this article is available at https://publons.com/publon/10.1002/brb3.3493.

## Data Availability

The data that support the findings of this study are available on request from the corresponding author.
